# A Decentralized, Academically Integrated Training Model for Rural General Practice in Japan: A Descriptive Program Evaluation

**DOI:** 10.1007/s11606-026-10469-5

**Published:** 2026-04-20

**Authors:** Kota Sakaguchi, Takeshi Endo, Yoshihiko Shiraishi, Makoto Kaneko, Takashi Watari

**Affiliations:** 1 General Medicine Center, Shimane University Hospital, Izumo City, Shimane Prefecture Japan; 2 Okuizumo Hospital, Shimane, Japan; 3 Oki Dozen Hospital, Shimane, Japan; 4 Department of Health Data Science, Yokohama City University, Yokohama, Japan; 5 Sagamihara Endowed Chair in Comprehensive Community Medicine, Kitasato University, Sagamihara, Japan; 6 Integrated Clinical Education Center, Kyoto University Hospital, Kyoto, Japan

## Abstract

**Background:**

Rural physician shortages persist globally, with professional isolation a major barrier. Traditional incentives have limited effects on long-term retention.

**Aim:**

To describe the decentralized, academically integrated training model at Shimane University General Medicine Center (SGMC) and evaluate recruitment and retention outcomes.

**Setting:**

Shimane Prefecture, a rural, aging region in Japan.

**Participants:**

Forty-two General Practice residents (2018–2025).

**Program Description:**

SGMC implemented a prefecture-wide “Neural GP Network” community of practice and a “Virtual Office” using Slack and Zoom. Residents completed longitudinal rural placements in local care teams.

**Program Evaluation:**

Annual recruitment averaged 15.8% of specialty trainees, significantly exceeding the national average (2.6%; *P* < 0.001). As of January 2026, 88.1% (37/42) remained in the prefectural network, exceeding estimates for rural Japan (~ 50%). The Virtual Office generated 26,787 messages and 6803 files (Feb 2024–Jan 2025). Since 2021, affiliates published 58 English-language papers.

**Discussion:**

Over 8 years, this hybrid model integrating digital networking with longitudinal training was associated with strong workforce stability, representing a promising approach to strengthen rural primary care.

**Supplementary Information:**

The online version contains supplementary material available at 10.1007/s11606-026-10469-5.

## INTRODUCTION

Shortages of primary care physicians in rural areas remain a global challenge, as financial incentives and compulsory service programs have shown only modest and often temporary effects on long-term retention.^1–3^ Japan faces similar difficulties: despite national redistribution policies, physician maldistribution persists, particularly in super-aging rural prefectures.^4^ General Practice (GP) was recognized as a new board-certified specialty only in 2018,^5^ and early-career clinicians frequently report professional isolation and limited access to academic mentorship as major barriers to sustained rural career commitment.^6,7^ These challenges suggest that the core problem in rural physician retention is not merely geographic or financial, but also motivational and relational in nature.

Qualitative studies from Japan have shown that young physicians working in remote rural settings experience substantial anxiety and professional isolation, particularly when practicing in relative solitude without adequate professional support.^8^ Building on these findings, we hypothesized that addressing psychological needs—specifically the sense of belonging, professional identity, and meaningful collegial connection—is a critical prerequisite for workforce retention, functioning as an essential complement to traditional financial or structural incentives.

Accordingly, in considering how to recruit physicians and enhance the training environment in depopulated rural areas where physician shortages are particularly severe, we drew on Self-Determination Theory (SDT), which posits that intrinsic motivation is sustained when the psychological needs of autonomy, competence, and relatedness are fulfilled.^9^ In parallel, the literature on communities of practice and professional identity formation emphasizes that belonging to a collegial, academically connected community is critical for maintaining engagement in rural practice.^3,10^ As the rationale for our reform, we judged that these needs are precisely what should be supported for early-career physicians. Based on this, we initiated a new program based on the premise that providing a training system explicitly structured around vertical, horizontal, and diagonal collaboration and grounded in face-to-face relationships in which individuals know one another would cultivate durable commitment to rural general practice beyond what financial incentives alone can achieve.

Against this backdrop, the Shimane University General Medicine Center (SGMC) developed a decentralized, academically integrated model featuring a Virtual Office, a prefecture-wide Neural GP Network, and longitudinal, community-embedded training. This report describes the design and early outcomes of this model, focusing on recruitment and early retention in comparison with national trends.

## SETTING AND PARTICIPANTS

The program operates in Shimane Prefecture, Japan, a predominantly rural region with a super-aging demographic in which more than one-third of the population is aged 65 years or older. Geographic diversity and degree of rurality across the prefecture are summarized using the Rurality Index for Japan (RIJ), a validated national measure ranging from 1 to 100 (Supplementary Figure S1).^4^ Among the seven secondary medical areas in Shimane Prefecture, four are classified as highly rural at the national level, and the remote Oki Islands medical area has an RIJ score of 92, indicating substantial geographic isolation and limited local healthcare capacity.

As the sole university hospital in the prefecture, Shimane University bears the primary responsibility for developing a regional physician workforce. Approximately 100 medical students graduate from Shimane University each year; of these, about 50 begin junior residency training within the prefecture, and approximately 30 remain for their specialty training there. Before the SGMC program was established in 2021, no board-certified general practitioners were formally affiliated with universities in Shimane Prefecture, underscoring the absence of an academic training pathway in rural general practice.

Participants in the SGMC program included GP residents and a growing network of rural general practitioners across 17 partner hospitals and clinics. These clinicians constitute the core membership of the Neural GP Network and engage in Virtual Office and community-embedded training.

## PROGRAM DESCRIPTION

The SGMC has developed a decentralized, academically integrated training system designed to mitigate professional isolation and strengthen rural primary care capacity across a prefecture-wide network. The model comprises three mutually reinforcing components: a Neural GP Network, the Virtual Office, and longitudinal community-embedded training.

1. The Neural GP Network (Structure)
  The term “Neural GP Network” is an expression inspired by the concept of neural networks, emphasizing organic, non-hierarchical connectivity among general practitioners, in which each clinician functions as an interdependent node within a dynamically evolving professional network. The Neural GP Network is a prefecture-wide community of practice that links more than 300 general practitioners and residents across 17 clinical sites. Rather than operating under a traditional hub-and-spoke model, it functions as a many-to-many peer-connected network that supports shared learning, real-time case discussions, and academic collaboration across geographically dispersed rural settings. Consistent with Self-Determination Theory (SDT), this structure is designed to strengthen professional identity and foster a sense of relatedness among clinicians who would otherwise practice in isolation.^9^ A conceptual overview is provided in Supplementary Figure S2.
2. The Virtual Office (Infrastructure)
  The Virtual Office provides a digital educational infrastructure that sustains day-to-day academic and clinical connectivity within the Neural GP Network. It comprises a secure Slack workspace for asynchronous communication and scheduled Zoom sessions for synchronous activities. Beyond ad hoc consultations, the Virtual Office functions as a structured, longitudinal educational environment.
  Specifically, it supports the following: (1) longitudinal mentorship relationships between GP residents and faculty generalists; (2) career coaching and professional development guidance; (3) regular case-based conferences and virtual teaching rounds; and (4) academic research support, including guidance on manuscript preparation and submission, alongside an on-demand video library that archives lectures, clinical case discussions, procedural demonstrations, and faculty-led educational sessions. These materials allow GP residents at geographically isolated sites to asynchronously access the same core educational content. Through this system, residents receive continuous supervision, feedback, and academic guidance, regardless of their physical location, effectively embedding them in an academically connected community of practice despite geographic dispersion.
3. Longitudinal, Community-Embedded Training (Activities)
  The GP residents completed longitudinal, community-embedded, and rural placements as integrated members of local care teams. This training design reflects the educational principle that continuity with patients, supervisors, and clinical contexts serves as a core organizing element in medical training.^11^ Prior international studies have shown that such longitudinal rural training exposure is strongly associated with subsequent rural practice and workforce retention.^12^ Activities span three domains:
  1. Clinical Practice: Providing comprehensive primary care under the supervision of local general practitioners.
  2. Mentoring and Academic Development: Supported through continuous online engagement with faculty and peers via the Neural GP Network and the Virtual Office.
  3. Community-Based Inquiry and Research: This involves enabling GP residents to identify local needs, lead projects, and cultivate autonomy and competence.
  A detailed overview of the learning objectives and core modules is provided in Supplementary Table S1.

## PROGRAM EVALUATION

### Evaluation Methods

We conducted a retrospective descriptive evaluation of the SGMC program using administrative records from the SGMC and the Shimane Prefecture Medical Association. National reference data on general practice recruitment were obtained from publicly available annual reports of the Japan Medical Specialty Board.^13^ The program population comprised 42 GP residents who entered the SGMC network between 2018 and 2025. We examined four domains of program-related indicators: recruitment, workforce retention, network engagement, and academic productivity.

Retention was defined as practicing within the Shimane prefectural network as of January 2026. For contextual comparison, the observed retention proportion was contrasted with the retention levels typically reported among physicians working in Japan’s most rural clinical settings (approximately 50%), as described in a recent national epidemiological study,^14^ using a chi-squared test. Network engagement was quantified using Virtual Office logs (Slack message volume and file-sharing activities) from February 2024 to January 2025, which served as a behavioral proxy for professional connectedness.

Academic productivity was assessed by enumerating the peer-reviewed English-language publications authored by SGMC-affiliated physicians during the study period. Publications were identified through a structured PubMed search using predefined queries targeting SGMC program faculty and Shimane University Hospital/SGMC affiliations. This approach was intentionally conservative and likely underestimated total scholarly output.

## RESULTS

### Participant Characteristics and Recruitment

**Figure 1:**
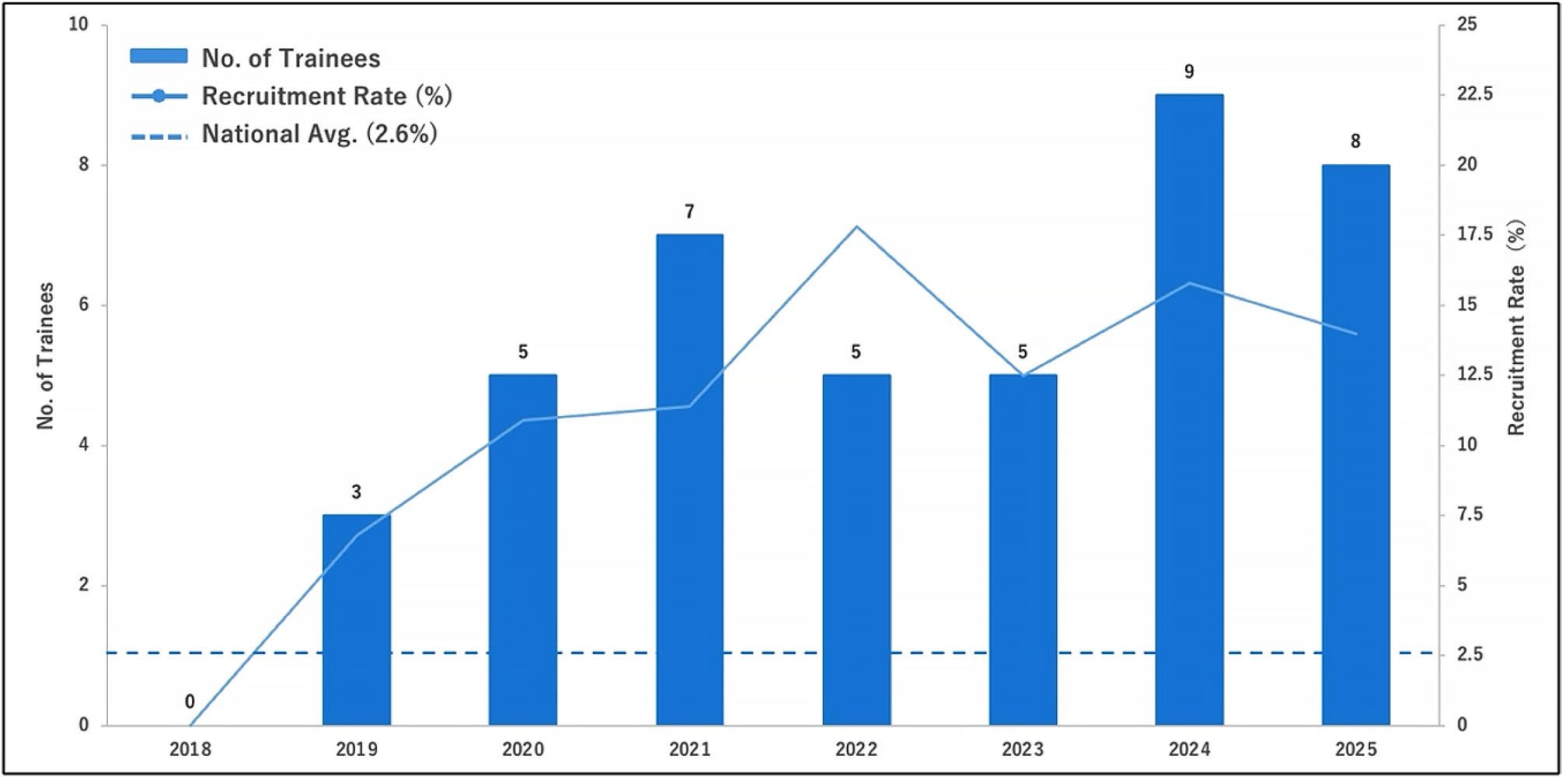
Trends in annual recruitment of General Practice residents in Shimane Prefecture compared with the national average (2018–2025). The bars represent the annual number of physicians entering the general practice training program at the Shimane University General Medicine Center. The solid line indicates the annual recruitment rate (% of all postgraduate residents) in Shimane Prefecture. The horizontal dashed line denotes the weighted mean national recruitment rate for general practice in Japan over the study period (2018–2025) (2.6%), calculated from the annual reports of the Japan Medical Specialty Board.^13^

A total of 42 GP residents entered the program between 2018 and 2025. As shown in Fig. 1, annual recruitment increased over time. The participant characteristics are summarized in Table 1. The median postgraduate year at entry was 3 (range, 3–8), reflecting the inclusion of both early- and mid-career practitioners. Notably, 40.5% (17/42) had graduated from medical schools outside the prefecture.

**Table 1:** Baseline Characteristics and Early Retention of Program Participants (2018–2025)

Characteristics	All participants (*N* = 42)
Career stage at entry	
PGY, median [range]^a^	3 [3–8]
Gender	
Male	28 (66.7%)
Female	14 (33.3%)
Medical school location (local vs non-local)^b^	
Shimane University (local)	25 (59.5%)
Other universities (non-local)	17 (40.5%)
Outcome (as of January 2026)	
Early retention within the regional network^c^	37 (88.1%)

*PGY*, postgraduate year

^a^Calculated based on years since medical licensure at the time of program entry. Values indicate the median and range (minimum–maximum) reflecting the inclusion of mid-career physicians

^b^Medical school location indicates undergraduate medical education. “Local” refers to graduates of Shimane University. “Non-local” refers to graduates of universities outside the prefecture

^c^Early retention is defined as a practice within the Shimane Prefecture regional network (including universities, community hospitals, and clinics) at the time of data collection (January 2026)

As of January 2026, 37 of these residents (88.1%) were practicing at SGMC-affiliated medical institutions within the prefectural network. This proportion was substantially higher than retention rates typically reported in the literature for physicians working in similar rural settings (approximately 50%) (*χ*^2^ = 24.4, *P* < 0.001).^14^

## Network Engagement and Academic Productivity

Over the latest 12-month observation period (February 2024 to January 2025), the Virtual Office, comprising 375 registered users, generated 26,787 messages and 6803 file exchanges. In terms of academic productivity, SGMC-affiliated physicians have produced 58 peer-reviewed English-language publications since the center was established in 2021. This output represents a marked increase compared to the pre-establishment period, during which international peer-reviewed publications were scarce.

## DISCUSSION

This report suggests that a decentralized and academically integrated training model may strengthen early recruitment and retention in rural general practice. The SGMC program was developed in response to persistent physician maldistribution in Japan and the well-documented challenge of professional isolation among rural clinicians. Our findings are consistent with the proposition that a training environment intentionally structured to support autonomy, competence, and relatedness, which make up the core components of Self-Determination Theory (SDT)^9^, may contribute to the strong early retention signal observed in this cohort.

The prefecture-wide Neural GP Network and the Virtual Office appear to address professional isolation by enabling a continuous many-to-many community of practices across geographically dispersed sites. Prior research from Australia, Canada, and Europe has highlighted that longitudinal exposure, strong professional identity formation, and academic connectivity are essential for sustainable rural workforce development.^1–3,6,10^ Our model aligns with these principles by introducing a digital infrastructure that facilitates real-time clinical dialogue, rapid peer consultation, and academic mentoring. The substantial volume of messaging and file-sharing within the Virtual Office suggests that digital platforms can meaningfully supplement traditional rural training models, particularly in regions where clinicians are widely distributed. However, we did not directly measure psychological constructs, such as perceived isolation, well-being, or burnout. Accordingly, online engagement should be interpreted as a behavioral indicator of professional connectedness rather than as evidence of changes in psychological outcomes.

The 88.1% retention rate observed at this early stage is encouraging relative to previously reported estimates in rural practice and is consistent with the literature suggesting that community-embedded, relationship-rich learning environments foster stronger intentions to remain in rural practice.^1,2,6^ Conventionally, efforts to recruit early career physicians and encourage practice in remote, depopulated rural areas are presumed to require structural and financial levers, such as higher compensation, the physical placement of supervising physicians, upgrades to hospital facilities and working conditions, and regulatory measures or policy interventions.^15,16^ However, as described above, our project was implemented in a context in which these conditions remained largely unchanged. This observation is important because it suggests that the observed improvements cannot be readily attributed to enhanced financial incentives or major structural reforms but rather to the programmatic components of the generalist network. Nevertheless, these findings warrant cautious interpretation. Although the program has been implemented over an 8-year period (2018–2025), the maximum follow-up duration for individual residents remains short in the context of a multi-decade professional career; therefore, long-term retention has not yet been determined. Additionally, the evaluation did not include economic analyses or cost-effectiveness assessments. Moreover, this descriptive analysis cannot establish causal relationships. Although the temporal association is suggestive, unmeasured contextual or systemic factors, such as concurrent national policy changes or local institutional dynamics, may also have contributed to the observed trends.

Despite these limitations, the SGMC program offers a potentially adaptable framework for rural workforce development. By integrating digital connectivity, decentralized rural training sites, and a theory-informed educational design, the program provides a replicable approach for regions facing similar demographic and geographic challenges. Future work will include the longitudinal tracking of alumni career trajectories and a mixed-method evaluation of the association between the network and psychological outcomes, including perceived isolation, well-being, and burnout, to better understand the long-term implications of this approach.

## Supplementary Information

Below is the link to the electronic supplementary material.

ESM 1 (DOCX 1.88 MB)

ESM 2 (DOCX 16.4 KB)

## Data Availability

The data presented in this study are available upon request from the corresponding author. The data are not publicly available because of privacy restrictions regarding program participants.

## Declarations

### Large Language Model (LLM) — Generative AI Use Statement

A large language model was used to assist with English-language editing for clarity and style. The model was not used to generate new content, analyze data, or influence scientific interpretation.
