# Supplementary material for: A Decentralized, Academically Integrated Training Model for Rural General Practice in Japan: A Descriptive Program Evaluation
Source: J Gen Intern Med. 2026 Apr 20;41(9):2618–23. doi: 10.1007/s11606-026-10469-5 (PMC13305047; doi:10.1007/s11606-026-10469-5)
Supplement: Supplementary file 1 — (DOCX 1.88 MB) [file 11606_2026_10469_MOESM1_ESM.docx]

**Supplementary Figure S1. Geographic distribution of the Rurality Index for Japan (RIJ) across Shimane Prefecture.**

**
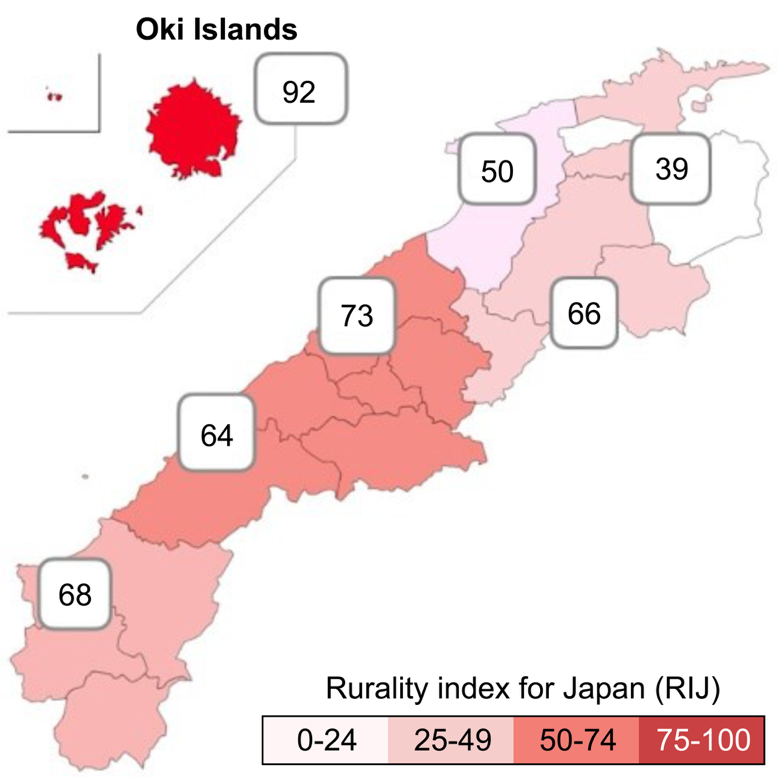
**

**Legend:**

The map illustrates the seven secondary medical areas of Shimane Prefecture. Areas are shaded according to their Rurality Index for Japan (RIJ) scores (range, 0–100), with darker shades indicating higher rurality. The Oki Islands (RIJ 92) represent the most geographically isolated region, whereas the central Izumo and Matsue areas show lower rurality scores. This geographic heterogeneity highlights the rationale for a decentralized training model.

**Supplementary Figure S2. Conceptual Diagram of the Neural GP Network**


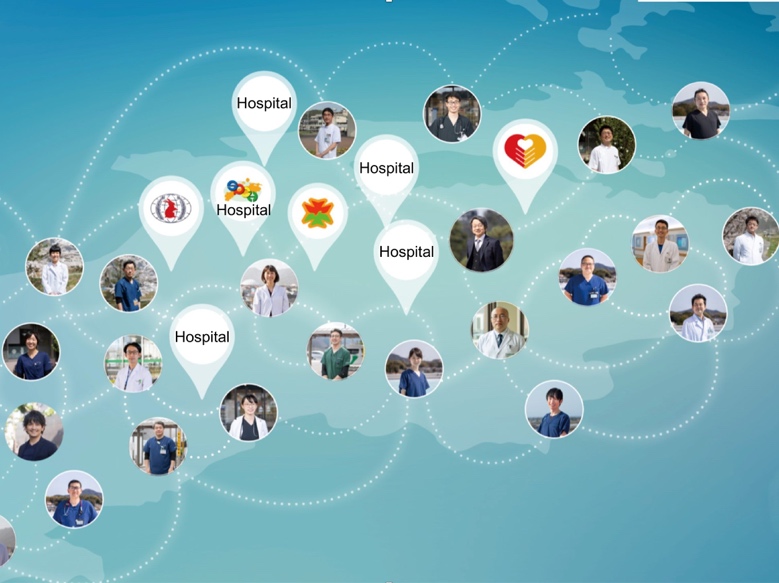


**Legend:**
This schematic illustrates the conceptual structure of the Neural GP Network, a prefecture-wide, many-to-many community of practice linking general practitioners, residents, and academic faculty across geographically dispersed rural sites in Shimane Prefecture. Unlike a hub-and-spoke model, the network emphasizes peer-to-peer connectivity, facilitating shared clinical learning, mentoring, and academic collaboration through both in-person interactions and the Virtual Office (Slack and Zoom). Connections shown are illustrative and intended to represent functional relationships rather than formal social network analysis.
