# Supplementary material for: A Decentralized, Academically Integrated Training Model for Rural General Practice in Japan: A Descriptive Program Evaluation
Source: J Gen Intern Med. 2026 Apr 20;41(9):2618–23. doi: 10.1007/s11606-026-10469-5 (PMC13305047; doi:10.1007/s11606-026-10469-5)
Supplement: Supplementary file 2 — (DOCX 16.4 KB) [file 11606_2026_10469_MOESM2_ESM.docx]

Supplementary Table S1. Overview of the SGMC Longitudinal Community-Embedded Training Curriculum

| Domain | Learning Objectives | Key Activities & Modules | Frequency / Duration |
| --- | --- | --- | --- |
| 1. Clinical Practice (Rural) | ・Develop competence in comprehensive rural general practice.  ・Manage undifferentiated acute and chronic conditions in resource-limited settings.  ・Practice patient-centered care with longitudinal continuity. | ・Rural Placement: Embedded clinical practice at rural clinics or small hospitals as a core member of the care team.  ・Home Care: Regular home visits for older adults and palliative patients.  ・Emergency Care: Participation in rural emergency care, including on-call duties. | ・Duration: Typically 3 years (aligned with the Japanese general practice residency program).  ・Intensity: Full-time or rotation-based placements, depending on individual training pathways. |
| 2. Mentoring & Academic Development | ・Form a strong professional identity as a generalist physician.  ・Acquire skills in evidence-based medicine (EBM) and clinical reasoning.  ・Maintain professional engagement and mitigate isolation through sustained peer and faculty interaction. | ・Virtual Office (Slack): Daily asynchronous case consultation and mentorship.  ・Zoom Conferences: Weekly or monthly synchronous case conferences (grand rounds, morning report, case report writing etc).  ・Career Coaching: Periodic one-on-one mentoring sessions with senior faculty or an external professional coach. | ・Daily: Slack-based interactions.  ・Weekly/Monthly: Scheduled online educational conferences.  ・Longitudinal: Continuous throughout the 3-year training period. |
| 3. Community-Based Inquiry & Research | ・Understand social determinants of health in rural communities.  ・Develop skills in community diagnosis and health promotion.  ・Cultivate autonomy and project management skills in real-world settings. | ・Community Diagnosis: Analysis of regional health data (e.g. administrative or public health databases).  ・Health Promotion Projects: Planning and leading local preventive care or community health initiatives.  ・Academic Writing: Writing case reports or conducting clinical research. | ・Project-based: Conducted longitudinally across the training period.  ・Expected Output: At least one academic presentation and/or at least one manuscript submission is required during the training period. |
